# The Antimicrobial Peptide MK58911-NH_2_ Acts on Planktonic, Biofilm, and Intramacrophage Cells of Cryptococcus neoformans

**DOI:** 10.1128/AAC.00904-21

**Published:** 2021-11-17

**Authors:** Junya de Lacorte Singulani, Lariane Teodoro Oliveira, Marina Dorisse Ramos, Nathália Ferreira Fregonezi, Paulo César Gomes, Mariana Cristina Galeane, Mario Sergio Palma, Ana Marisa Fusco Almeida, Maria José Soares Mendes Giannini

**Affiliations:** a Department of Clinical Analysis, School of Pharmaceutical Sciences, São Paulo State University-UNESP, Araraquara, Brazil; b Department of Basic and Applied Biology, Institute of Biosciences, São Paulo State University-UNESP, Rio Claro, Brazil

**Keywords:** antimicrobial peptide, systemic mycoses, antifungal activity, cryptococcosis, biofilm, nonconventional animal models, intramacrophage activity, *Cryptococcus neoformans*, antifungal agents, mechanisms of action, systemic fungi

## Abstract

Cryptococcosis is associated with high rates of morbidity and mortality, especially in AIDS patients. Its treatment is carried out by combining amphotericin B and azoles or flucytosine, which causes unavoidable toxicity issues in the host. Thus, the urgency in obtaining new antifungals drives the search for antimicrobial peptides (AMPs). This study aimed to extend the understanding of the mechanism of action of an AMP analog from wasp peptide toxins, MK58911-NH_2_, on Cryptococcus neoformans. We also evaluated if MK58911-NH_2_ can act on cryptococcal cells in macrophages, biofilms, and an immersion zebrafish model of infection. Finally, we investigated the structure-antifungal action and the toxicity relationship of MK58911-NH_2_ fragments and a derivative of this peptide (MH58911-NH_2_). The results demonstrated that MK58911-NH_2_ did not alter the fluorescence intensity of the cell wall-binding dye calcofluor white or the capsule-binding dye 18b7 antibody-fluorescein isothiocyanate (FITC) in C. neoformans but rather reduced the number and size of fungal cells. This activity reduced the fungal burden of C. neoformans in both macrophages and zebrafish embryos as well as within biofilms. Three fragments of the MK58911-NH_2_ peptide showed no activity against Cryptococcus and not toxicity in lung cells. The derivative peptide MH58911-NH_2_, in which the lysine residues of MK58911-NH_2_ were replaced by histidines, reduced the activity against extracellular and intracellular C. neoformans. On the other hand, it was active against biofilms and showed reduced toxicity. In summary, these results showed that peptide MK58911-NH_2_ could be a promising agent against cryptococcosis. This work also opens a perspective for the verification of the antifungal activity of other derivatives.

## INTRODUCTION

In recent years, there has been a substantial increase in the incidence of systemic mycosis due to both medical advances and the rise of immunosuppressive diseases. Cryptococcosis, for example, was estimated to be responsible for more than 200,000 incident cases worldwide in 2014 and 15% of AIDS-related deaths (1). Its etiological agents are found in environments contaminated by feces of pigeons and other birds (Cryptococcus neoformans) or by the decomposition of wood and other plant materials (Cryptococcus gattii) (2).

The first site of infection of Cryptococcus spp. is the lung, where alveolar macrophages are the first immune cells to encounter the fungus. In this organ, either the host’s immune response controls the infection or the fungus successfully escapes this response and, without treatment, spreads systemically, with fatal consequences. In order to disseminate within the host, Cryptococcus spp. can proliferate, transfer, and escape from macrophages lytically or nonlytically (via vomocytosis) (3–5). This process is believed to strongly influence the migration of C. neoformans and C. gattii to the central nervous system, where they cause cryptococcal meningitis in immunocompromised patients, especially those in advanced stages of AIDS; patients on immunosuppressive therapy; or patients with hematological malignancies (6–8).

Although rapid antifungal therapy is relatively effective, there is a limited number of classes of these drugs. Cryptococcosis is usually treated with fluconazole, while severe cases, including cryptococcal meningitis, are treated with amphotericin B together with 5-fluorocytosine or fluconazole (9). However, many antifungal agents display side effects and drug-drug interactions (10). Another problem is increasing fungal species with natural or acquired resistance to currently available antifungal drugs. For example, C. neoformans has intrinsic resistance to a newer class of antifungal agents, the echinocandins (11, 12). In addition, Cryptococcus spp. can colonize and form biofilms on medical devices such as ventriculoatrial shunt catheters, prosthetic valves, and prostheses, representing a significant health concern due to their resistance to antifungal treatment (13). Thus, the search for new antifungal targets and more efficient and less toxic drugs has been ongoing.

Antimicrobial peptides (AMPs) are important molecules that act as the first line of defense to combat pathogens in many organisms and are attractive alternatives to antifungal drugs. Some AMPs have multiple targets in microorganisms, which reduces the chances of resistance development, while others are more specific in their mechanism of action, acting mainly on the bacterial and/or fungal membrane (14, 15). In addition, their applications are also being investigated as promising antibiofilm drugs (16, 17).

Here, we explore a social wasp venom-derived AMP, which belongs to the mastoparan class, as a promising antifungal agent. Previous studies with mastoparan peptides revealed their activity against Gram-positive and Gram-negative bacteria through interaction with membranes (18, 19). Furthermore, we demonstrate that the mastoparan derivative MK58911-NH_2_ causes membrane damage in C. neoformans cells and has *in vivo* antifungal efficacy in the Galleria mellonella wax moth model (20).

Here, we provide more detailed information about the mode of action of MK58911-NH_2_ and verify its efficacy against C. neoformans in macrophages and biofilms. We also evaluate if fragments from MK58911-NH_2_ and its derivative MH58911-NH_2_, in which the lysine residues of MK58911-NH_2_ were replaced by histidine residues, still present antifungal activity and toxicity. For *in vivo* assays, we used zebrafish embryos, which have been applied extensively for toxicological studies in drug development (21) and more recently as a model for fungal infections, including Cryptococcus spp. (22–25).

## RESULTS

**FIG 1:**
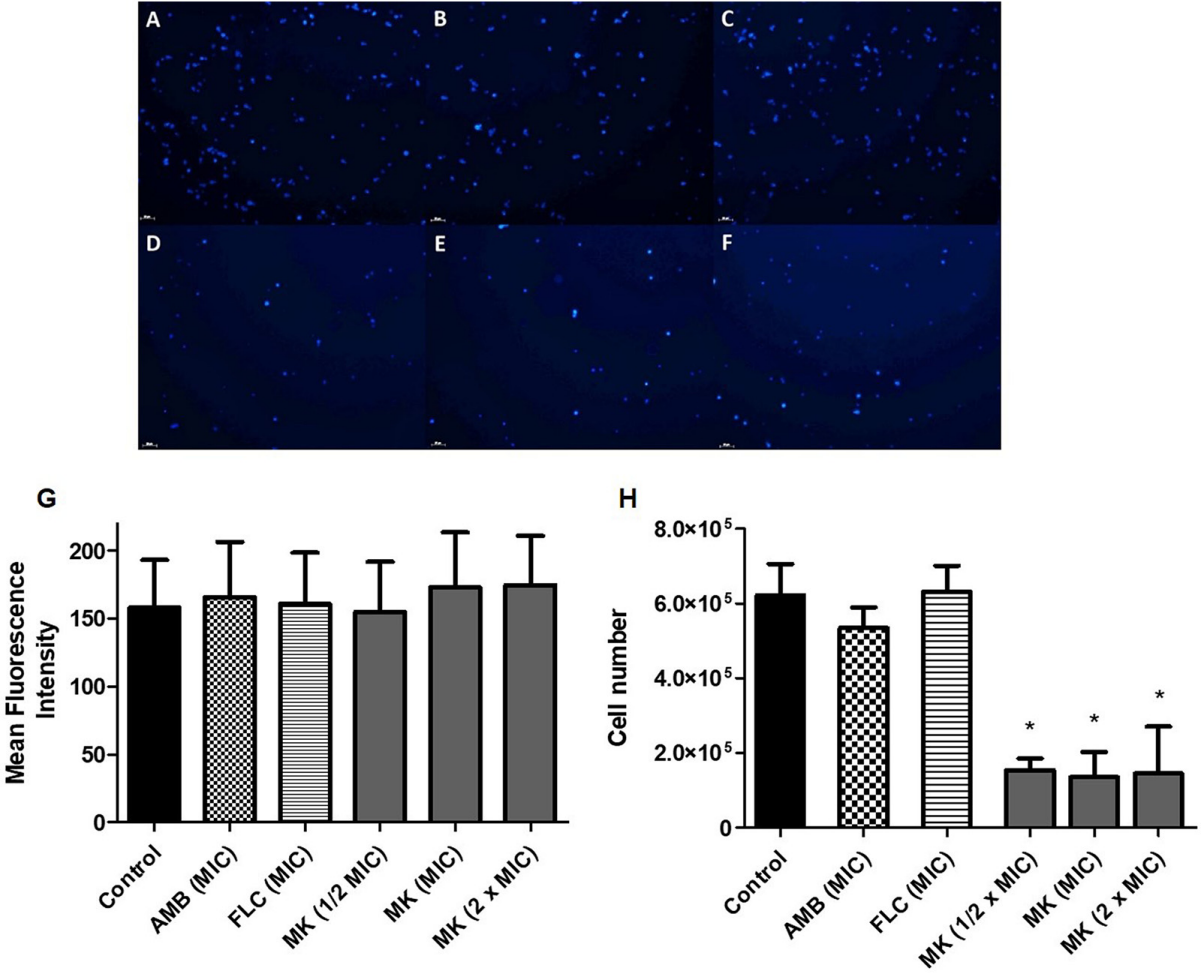
C. neoformans cells exposed to the peptide for 4 h and marked with calcofluor white. (A) Control; (B) amphotericin B (AMB) at the MIC; (C) fluconazole (FLC) at the MIC; (D to F) peptide MK58911-NH_2_ (MK) at 1/2× MIC, the MIC, and 2× MIC; (G) average fluorescence intensity per cell; (H) number of cells. Data represent the means (± standard deviations [SD]) from three independent experiments. *, *P* < 0.05 compared to the control.

The intensity of calcofluor white fluorescence of C. neoformans cell walls did not reveal a change in the chitin content upon exposure to MK58911-NH_2_ (Fig. 1D to G) compared to the control (Fig. 1A and G) or cells treated with amphotericin B (Fig. 1B and G) and fluconazole (Fig. 1C and G) after 4 h of treatment. On the other hand, the number of cells in the control group, 6.2 × 10^6^ (Fig. 1A and H), was significantly reduced to 1.4 × 10^6^ cells (*P* < 0.05) (Fig. 1D to F and H) in the group incubated in the presence of MK58911-NH_2_.

**FIG 2:**
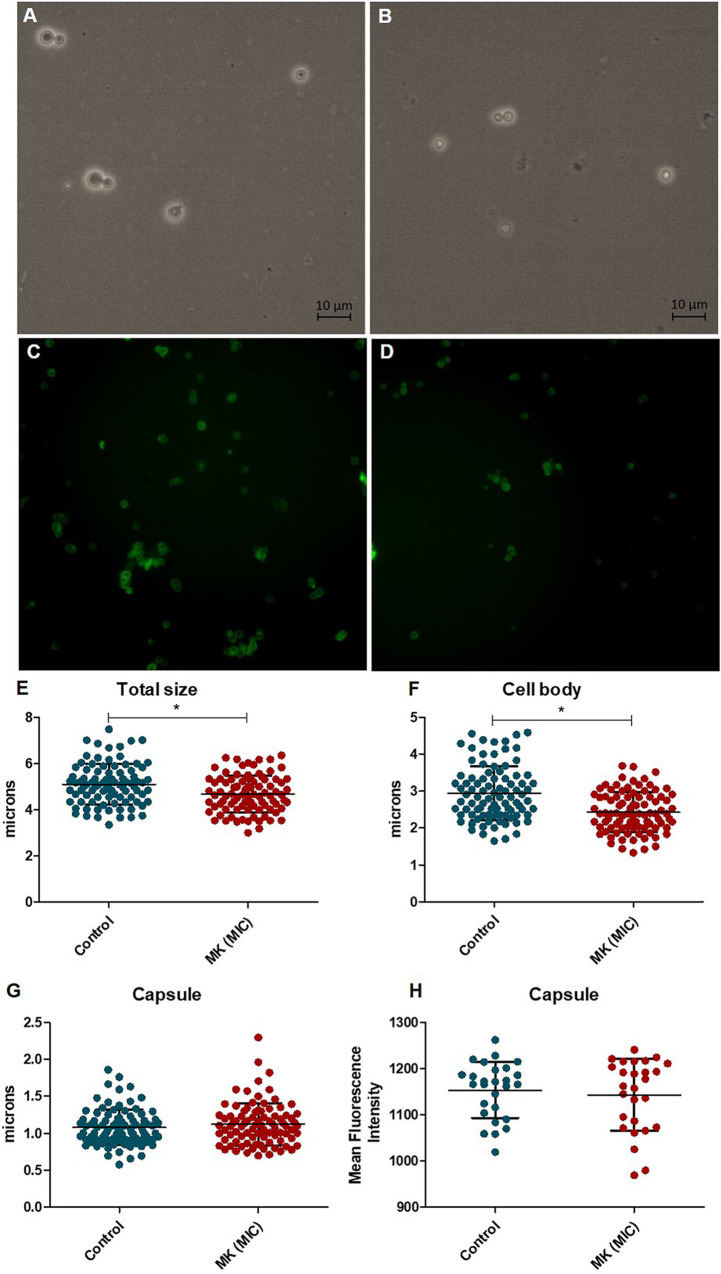
C. neoformans cells exposed to the peptide for 4 h and marked with India ink (A, B, and E to G) or antibody 18b7 (C, D, and H). (A and C) control; (B and D) peptide MK58911-NH_2_ (MK) at the MIC; (E) total cell diameter; (F) diameter of the cell body (without capsule); (G) diameter of the capsule; (H) average fluorescence intensity per cell. Data represent the means (±SD) from three independent experiments. *, *P* < 0.05 compared to the control.

Staining the capsule with India ink revealed that MK58911-NH_2_ did not change the diameter of this structure compared to the untreated control group (Fig. 2A, B, and G). However, the peptide reduced the total and body diameters (without capsule) of the fungal cell (*P* < 0.05) (Fig. 2A, B, E, and F). Staining C. neoformans with antibody 18b7-fluorescein isothiocyanate (FITC) demonstrated no difference in the mean fluorescence intensities between the capsules of C. neoformans cells treated with the peptide and those of the control group (Fig. 2C, D, and H).

**FIG 3:**
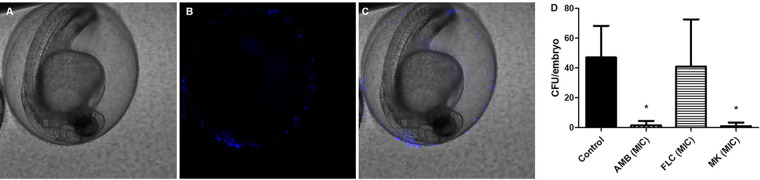
Representative images of zebrafish embryos exposed to a fungal suspension (1 × 10^6^ cells/ml) of C. neoformans. (A) Bright-field image; (B) fungal cells labeled with calcofluor white; (C) overlay of images; (D) effect on the fungal load in embryos infected and exposed to treatments with MK58911-NH_2_ (MK), amphotericin B (AMB), and fluconazole (FLC) at the MIC for 24 h. Data represent the means (±SD) from two independent experiments. *, *P* < 0.05 compared to the control.

The peptide action was also verified in zebrafish embryos exposed to a fungal suspension of C. neoformans at 1 × 10^6^ cells/ml for 24 h using an immersion model. During this period, fungal cells adhered to the embryos’ chorion, for which the images of cells (labeled with calcofluor white) were acquired 4 h after exposure to the fungus (Fig. 3A to C). Exposure to treatments with MK58911-NH_2_ or the control drug amphotericin B for 24 h resulted in a significant reduction in CFU compared to the untreated control (Fig. 3D). In contrast, the drug fluconazole did not reduce the fungal burden of the embryos’ chorion. All embryos survived treatment with MK58911-NH_2_ or antifungal drugs.

The peptide MK58911-NH_2_ can also be an excellent model for designing analog peptides as novel antifungal agents. In this aspect, four derivative peptides from MK58911-NH_2_ were synthesized and used to analyze antifungal activity and cytotoxicity. In one of these peptides, the lysine residues were replaced by histidine residues, while the other amino acid residues were maintained in the same positions as those observed in MK58911-NH_2_; this peptide was designated MH58911-NH_2_ (Table 1). Meanwhile, the other three peptides corresponded to shorter sequences of the MK58911-NH_2_ peptide to verify which fragments could be responsible for the activity. Furthermore, to establish a correlation between the structural features of the peptides and the biological activities, we simulated the secondary structures of each peptide using the PEP2D algorithm as a bioinformatic tool (26). The results are shown in Table 1, which reveals that MK58911-NH_2_ presents 57.14% helices and 42.86% coils; meanwhile, MH58911-NH_2_ presents 50% helices and 50% coils. The simulation for MK58911(4–11)-OH revealed 37.50% helices and 62.50% coils. The algorithm calculated that MK58911(1–7)-OH presents one element of the sheet, while MK58911(8–14)-NH_2_ has two of these elements of secondary structure, covering 14.29% and 28.57% of their sequences, respectively. The remaining structures of these peptides are constituted of coils. However, *in vitro* assays revealed that none of the peptide fragments or derivatives showed activity against Cryptococcus spp. and toxicity to pulmonary fibroblasts (MRC5 cells) at the evaluated concentrations (Table 2).

**TABLE 1:**
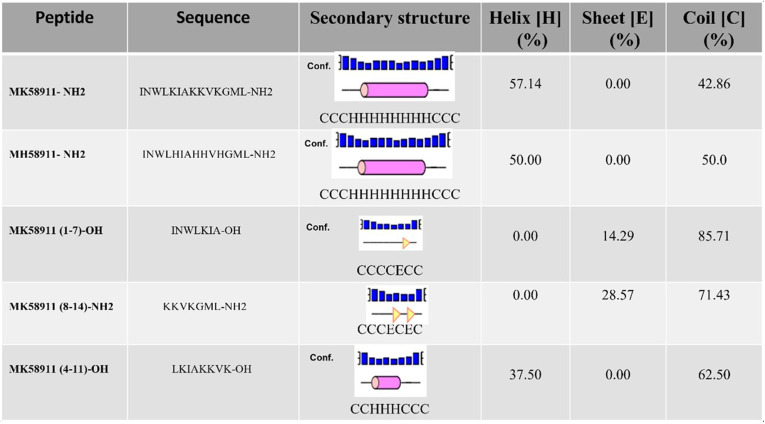
Amino acid sequences and prediction of secondary structures of the peptides tested in the present study^*a*^

a Prediction of the secondary structures of the peptides was based on the PEP2D algorithm (https://webs.iiitd.edu.in/raghava/pep2d/submit.html). The structural elements are represented as follows: helix, pink cylinders; sheet, yellow triangles; coil, black lines.

**TABLE 2:** Activity against Cryptococcus species and toxicity in pulmonary fibroblasts (MRC5 cells) of MK58911-NH_2_ peptide derivatives^*a*^

Peptide or drug	C. neoformans MIC (μg/ml)	C. gattii MIC (μg/ml)	MRC5 IC_50_ (μg/ml)
MK58911-NH_2_	31.25	15.63	>500
MK58911(1–7)-OH	>250	>250	>500
MK58911(8–14)-NH2	>250	>250	>500
MK58911(4–11)-OH	>250	>250	>500
MH58911-NH_2_	>250	>250	>500
Amphotericin B	0.125	0.125	
Fluconazole	1	8	

a IC_50_, 50% inhibitory concentration.

**FIG 4:**
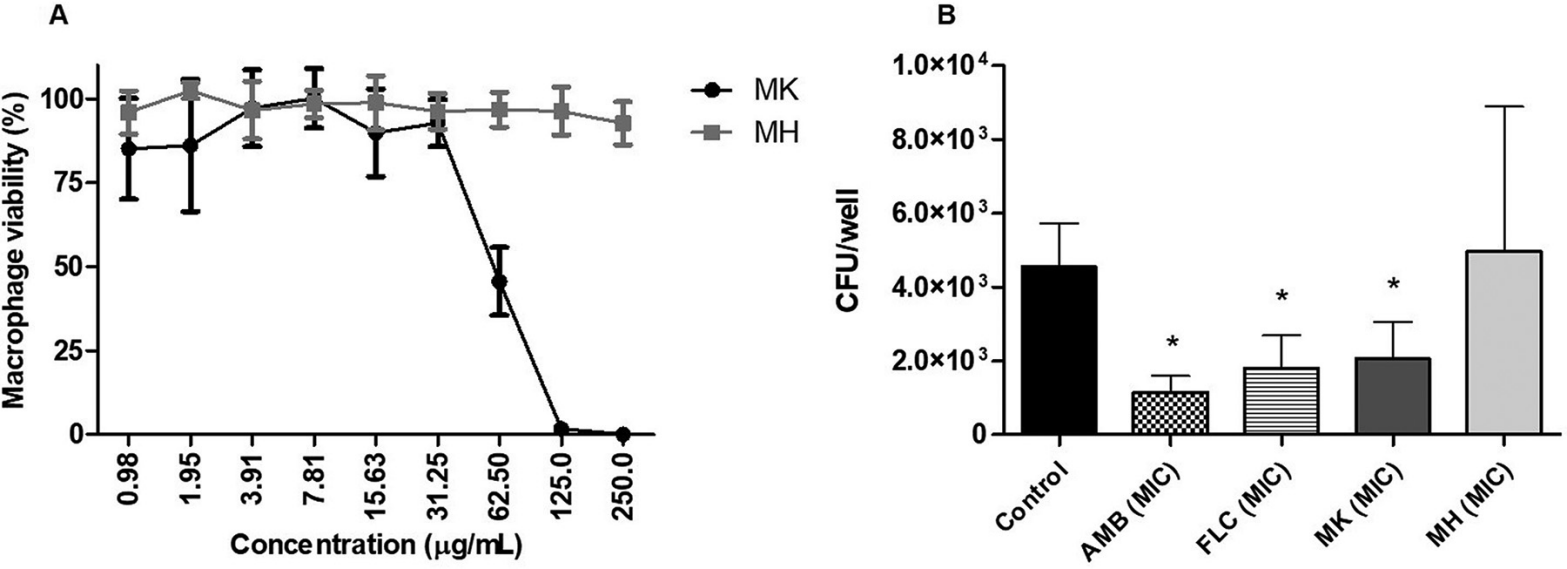
Activity of peptides MK58911-NH_2_ and MH58911-NH_2_ against intramacrophage C. neoformans (A) and on the viability of J774 macrophages (B). AMB, amphotericin B; FLC, fluconazole. Data represent the means (±SD) from three independent experiments. *, *P* < 0.05 compared to the control.

The determination of the activity of the peptides MK58911-NH_2_ and MH58911-NH_2_ against C. neoformans within macrophages and biofilms and their comparison were also performed. The J774 macrophage line was infected with C. neoformans and then exposed to treatments with the peptides and the control drugs amphotericin B and fluconazole. As shown in Fig. 4A, the peptide MK58911-NH_2_, amphotericin B, and fluconazole led to a significant reduction (*P* < 0.05) in the intramacrophagic fungal burden, with averages of 2.0 × 10^3^, 1.1 × 10^3^, and 1.8 × 10^3^ CFU/well, respectively, compared to the control (4.5 × 10^3^ CFU/well). However, the cells treated with MH58911-NH_2_ showed an average CFU value (4.9 × 10^3^ CFU/well) similar to that of the control. The cytotoxicity of both peptides was also verified with macrophages, and MH58911-NH_2_ was shown to be less toxic, with a 50% inhibitory concentration (IC_50_) of >250 μg/ml, than MK58911-NH_2_, with an IC_50_ of 62.50 μg/ml (Fig. 4B).

**FIG 5:**
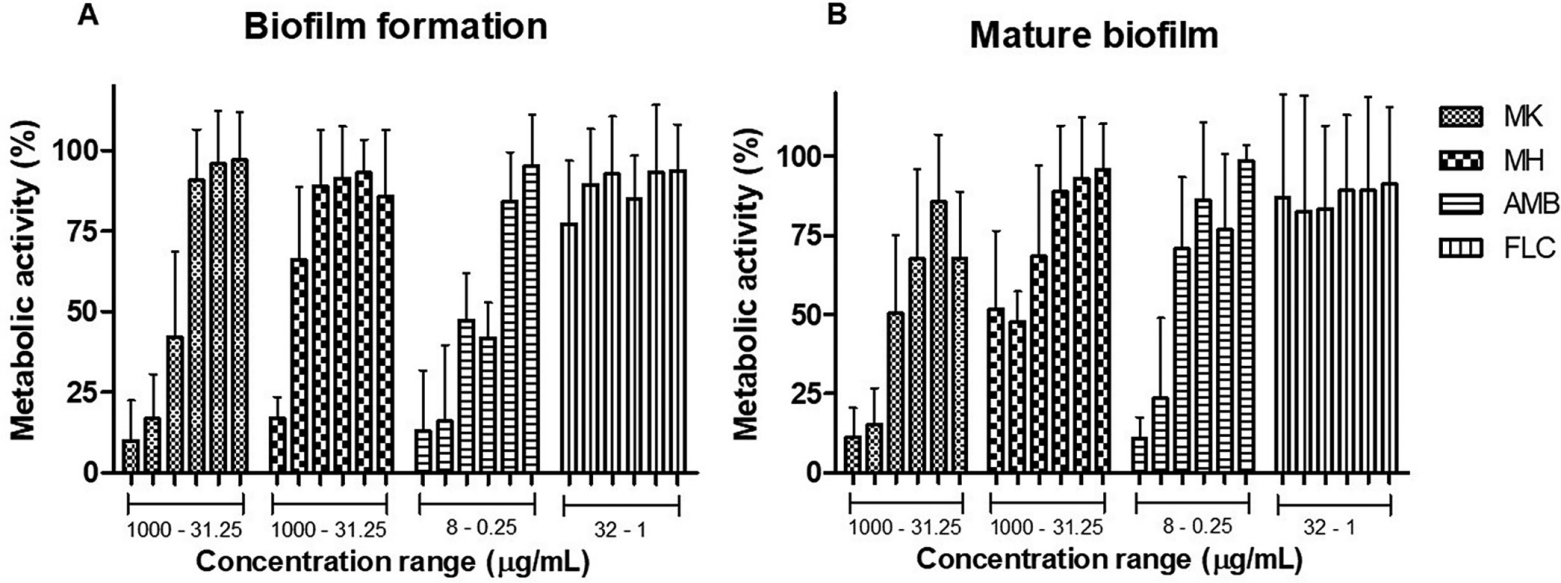
Activity of peptides MK58911-NH_2_ (MK) and MH58911-NH_2_ (MH) on biofilm formation at 0 h (A) and mature biofilm at 48 h (B) of C. neoformans. AMB, amphotericin B; FLC, fluconazole. Data represent the means (±SD) from three independent experiments. *, *P* < 0.05 compared to the control.

To test activity against biofilms of C. neoformans, treatments were carried out at 0 h (the beginning of biofilm formation) and on fully formed biofilms at 48 h. Both peptides decreased the metabolic activity of biofilms in a concentration-dependent manner, where values of ≥250 μg/ml for MK58911-NH_2_ and ≥500 μg/ml for MH58911-NH_2_ led to a reduction of at least 50% of the metabolic activity of both nascent (Fig. 5A) and mature (Fig. 5B) biofilms.

## DISCUSSION

Considering the inferior status of currently available antifungal drugs, new treatment options for cryptococcosis are needed. In this respect, studies from several groups have attempted to address the use of AMPs as antifungal agents (15). Currently, more than 3,000 AMPs are known; however, the mechanisms of action are characterized for only a reduced number of these peptides (27). The interaction of the AMPs with the fungi generally targets the fungal cell membrane and/or cell wall; however, some peptides can interact with intracellular targets (28). We previously demonstrated the *in vitro* and *in vivo* activity of the MK58911-NH_2_ peptide against Cryptococcus and its mechanism of action on the fungal membrane (20). Here, we verified the possible action of peptides on other cell structures of C. neoformans.

The cryptococcal cell wall comprises glucans (more β-1,6- than β-1,3-glucan), chitin, chitosan, mannoproteins, and glycosylphosphatidylinositol (GPI)-anchored proteins. Collectively, these components maintain the shape and rigidity of the cell and are essential for infection (29, 30). The polysaccharide capsule is anchored to the outer layer of the cell wall and is considered the main virulence factor of Cryptococcus spp. (29–31). The capsule of this yeast is composed of glucuronoxylomannan (GXM), galactoxylomannan (GalXM), and mannoproteins and is important for protection from desiccation, radiation, and phagocytosis (23, 31–34). All elements of the fungal cell wall and capsule can be excellent targets for antifungal compounds. However, our peptide MK58911-NH_2_ did not alter the fluorescence intensity of calcofluor white (chitin-binding dye) and 18b7-FITC (GXM-binding antibody) staining of the cell wall and capsule, respectively, in C. neoformans; on the other hand, this peptide reduced cell size and led to fungal death, which might be a result of cell membrane damage (20, 35, 36). The peptides from the mastoparan class such as MK58911-NH_2_ seem to accumulate on the membrane of microorganisms in a carpet-like manner, attracted by electrostatic interactions, and consequently, the membranes are disrupted (18, 37).

We next assessed the effect of MK58911-NH_2_ on intramacrophage C. neoformans. Although most studies have focused on compounds that act on extracellular cryptococci, the action on intracellular fungi is very important since the replication and spread of the host’s fungi are partially determined by macrophage-pathogen interactions (38). In our study, the peptide MK58911-NH_2_ reduced the intramacrophage fungal burden, in addition to exhibiting a direct effect on the fungus, as previously described (20).

Due to the pronounced risk of antifungal resistance associated with biofilm formation, the effect of MK58911-NH_2_ on the initial stage and mature biofilms was also evaluated. The resistance of fungal biofilms involves several features such as the overexpression of drug resistance genes and impairment of drug penetration due to the extracellular matrix (39). This study produced results that corroborate the findings of Martinez and Casadevall (13), which demonstrated that C. neoformans biofilms are resistant to fluconazole. However, the formation of C. neoformans biofilms can be prevented at concentrations of >0.5 μg/ml, and mature biofilms can be inhibited at concentrations of >2 μg/ml by high doses of amphotericin B. MK58911-NH_2_ presented an antifungal effect on different phases of the biofilm process at formation and mature biofilms. Furthermore, higher concentrations than those in planktonic cells were required for this peptide action (8× MIC), which are lower concentrations than expected for antifungal agents (100× to 1,000× MIC) according to previous studies (39, 40). Similar to MK58911-NH_2_, another peptide from the mastoparan class, polybia-MPI, caused membrane damage and inhibition of biofilm formation of *Candida* species (41).

After *in vitro* assays, we used an immersion model of zebrafish embryos for C. neoformans adhesion and evaluation of peptide antifungal efficacy. Treatment with MK58911-NH_2_ resulted in a significant reduction in the burden of fungi adhered to the chorion of the embryos. The noninvasive zebrafish embryo bath immersion model was performed using Candida albicans, in which the medium used, the inoculum concentration, and the ideal incubation time for the experiment were standardized (22). Subsequently, it was demonstrated that the adhered fungal cells form biofilms on the embryos’ chorion (42). Although we verified that a smaller number of C. neoformans cells adhered to the embryos than that of C. albicans cells demonstrated in previous studies (22, 42), the structure observed in our analysis is biofilm-like, with apparent agglomerate and extracellular matrix formation at 24 h postinfection (data not shown). Another result that corroborates this fact is that fluconazole did not reduce the fungal burden in the embryos, which also happens when this drug is tested against C. neoformans biofilms *in vitro* (13). However, further studies are needed to confirm this suggestion.

Finally, we tested some fragments of the peptide MK58911-NH_2_ to try to find out which portion of the molecule is responsible for its antifungal activity. A derivative with the same sequence as that of MK58911-NH_2_ but in which the lysine residues have been replaced by histidine residues (MH58911-NH_2_) was also assayed. In an attempt to correlate the structural features of the MK and MH series of peptides with their biological activities, the secondary structures of the five peptides used in the present study were simulated using a bioinformatics approach. AMPs are typically constituted of up to 54 amino acid residues in their sequences, presenting mostly net positive charges at physiological pH (43). Considering their secondary structures, the AMPs may be classified as (i) linear α-helical peptides, (ii) α-helical peptides presenting a disulfide bridge in the C-terminal region, (iii) β-sheet peptides presenting disulfide bridges, (iv) cyclic peptides, and (v) peptides rich in a specific amino acid residue. The α-helical peptides generally do not present a well-defined secondary structure in aqueous medium but fold into amphiphilic α-helices in the presence of the hydrophobic environment of cell membranes along the helical axis (44).

The results showed that none of the fragments of the peptide MK58911-NH_2_ showed anticryptococcal activity and toxicity to pulmonary fibroblasts (MRC5 cells) at the evaluated concentrations. Despite MK58911(4–11)-OH presenting a helix element of secondary structure, the peptide length does not seem to be enough to make this peptide active as an antifungal compound. The two other peptides [MK58911(1–7)-OH and MK58911(8–14)-NH_2_] do not present anticryptococcal activity. These results suggest that some smaller fragments lack all or part of the helical element, which is required for antifungal activity. The removal of some residues from any of the termini apparently breaks the α-helical conformation necessary to promote the reported biological actions.

Regarding the comparison of the antifungal activities of MK58911-NH_2_ and its derivative MH58911-NH_2_, it was observed that the lysine-containing peptide was more potent than the histidine-containing one. This result may be related to the fact that at physiological pH (used in the assay), the peptide carrying lysine residues presents the Lys side chains protonated, while in the peptide carrying histidine residues, the His side chains (imidazole) are neutral at physiological pH. Thus, the polycationic nature of MK58911-NH_2_ is more pronounced than that presented by MH58911-NH_2_ at physiological pH, making the lysine peptide more active. Following the present findings, a previous study demonstrated that the replacement of all basic amino acids in the heparin-binding peptides ARK24 and AKK24 by histidines (AHH24:1 and AHH24:2) abrogates their antibacterial and antifungal activities (45). However, the derivative MH58911-NH_2_ showed less toxicity in macrophages than MK58911-NH_2_, demonstrating that the insertion of histidine residues is beneficial for this parameter. The histidine residues present reduced pK_a_ values compared to lysine residues; thus, despite maintaining polycationicity, the total replacement of lysine by histidine modifies the biophysical parameters of the molecule, which in turn causes the reduction of the toxicity of MH58911-NH_2_ in macrophages.

We also evaluated the effect of MH58911-NH_2_ on C. neoformans in macrophages and biofilms. We hypothesize that although this peptide had no action against Cryptococcus spp., the pH value at which histidines ionize (gaining a positive charge) is lower than the pH value at which lysines ionize. In this way, the more acidic medium of the interior of the macrophages could benefit MH58911-NH_2_ activity, as demonstrated by a previous study with histidine-rich peptides (45). However, the peptide was unable to act on intramacrophage fungi. On the other hand, MH58911-NH_2_ at higher concentrations exhibited activity against C. neoformans in biofilms. Recent reviews (16, 46) demonstrated that some AMPs act against microorganisms in both planktonic and biofilm forms, such as MK58911-NH_2_. Others do not demonstrate activity against planktonic cells but can act in biofilms by the rupture or alteration of extracellular matrix components, which is a possible mechanism of MH58911-NH_2_.

In conclusion, the peptide MK58911-NH_2_ did not promote damage to the cell wall and capsule of C. neoformans but reduced the number and size of fungal cells. In addition, it reduced the fungal burden of C. neoformans in macrophages and zebrafish embryos. It also acted on biofilm formation and mature biofilms of C. neoformans. A derivative peptide (MH58911-NH_2_) in which the lysine residues of MK58911-NH_2_ were replaced by histidine residues reduced the activity against extracellular and intracellular C. neoformans. On the other hand, it was active against biofilms, and it was beneficial in reducing toxicity in macrophages. In summary, the results *in vitro* and *in vivo* show that peptide MK58911-NH_2_ can be a promising agent against cryptococcosis.

The present work opens a perspective for continuing peptide tests in mammalian models and verifying the antifungal activity and toxicity of other fragments and derivatives.

## MATERIALS AND METHODS

### Fungal strains.

C. neoformans ATCC 90112 and C. gattii ATCC 56990 were purchased from the collection of the Laboratory of Clinical Mycology, Faculty of Pharmaceutical Sciences of UNESP, Araraquara, Brazil. Cryptococcus species were grown at 30°C for 48 to 72 h in Sabouraud dextrose agar.

### Peptides.

The peptides were synthesized under a solid phase using an automatic synthesizer (Prelude model; Protein Technologies Inc., USA), based on a 9-fluorenylmethoxy carbonyl (Fmoc) strategy, with the *N*-9-fluorenylmethoxy carbonyl reagent. The peptides were synthesized using 100 mg of NovaSyn TGR resin, with a degree of substitution of 0.2 mmol/g. In each cycle of synthesis, Fmoc-amino acid-OH (Novabiochem), containing *N*-hydroxybenzotriazole (HOBt·H_2_O; Novabiochem) and *N*-methylmorpholine (NNM; Aldrich), was added as the amino acid-activating agent, and benzotriazol-1-1-hexafluorophosphate-oxy-Tris-pyrrolidine-phosphonium (PyBOP; Novabiochem) was added as a coupling agent, for 30 min. After each coupling cycle, five washes of the resin were performed with *N*,*N*-dimethylformamide (DMF; Sigma). Fmoc removal was performed using 30% (vol/vol) piperidine (Sigma) in DMF. All reactions were performed under mechanical agitation in a safety chapel. After coupling the last amino acid residue, the resin was washed with methanol (Mallinckrodt) and dried in a lyophilizer (model MLW-LGA 05; Heto). After drying, cleavage between the peptide and resin was performed using a solution containing 82.5% (vol/vol) trifluoroacetic acid (TFA; Mallinckrodt), 5% (vol/vol) anisole (Sigma), 2.5% (vol/vol) ethanedithiol (Aldrich), 5% (mass/vol) phenol, and 5% (vol/vol) ultrapure water for 2 h under mechanical stirring. This TFA-anisol-ethanedithiol-phenol-peptide solution was filtered to remove the resin and centrifuged at 4°C (model 5810R; Eppendorf) for 15 min at 3,000 rpm in the presence of ethyl ether (Synth). The sedimented material was then resuspended in ultrapure MilliQ Advantage A10 water (Millipore) and purified by reverse-phase high-performance liquid chromatography (RP-HPLC). The quality control for the peptides was done by electrospray ionization-mass spectrometry (ESI-MS) analysis.

The mastoparan analog peptide MK58911-NH_2_ (MK) was previously engineered and used as a structural model for the design of other analog peptides (19). Three fragments of the peptide MK58911-NH_2_ were also synthesized: MK58911(1–7)-OH (the C-terminal half), MK58911(8–14)-NH_2_ (the N-terminal half), and MK58911(4–11)-OH (the central portion) (Table 1). Furthermore, an analog peptide of MK58911-NH_2_ was synthesized in which all the lysine residues were replaced by histidine residues (MH58911-NH_2_). Fresh aqueous solutions in sterile water were prepared for all peptides to be used in the bioassays.

### Simulation of the secondary structures of peptides.

The sequences of the five peptides were loaded into the dialog box of the PEP2D algorithm (https://webs.iiitd.edu.in/raghava/pep2d/submit.html) (26) and submitted to estimate the occurrence of elements of secondary structures using the RandomForest classifier approach, which calculates the probabilistic values of the helices, sheets, and coils. Next, values are optimized using weights, and finally, the probability of each element of the secondary structure is assigned. The results were shown in a table format, exhibiting the percentage of each element of secondary structure and a graphic representation of the secondary structures for each peptide.

### Cell wall and fungal cell counts.

C. neoformans colonies were dissolved in phosphate-buffered saline (PBS) and diluted in a solution containing RPMI 1640 culture medium, 0.165 M morpholinepropanesulfonic acid (MOPS) (pH 7.4), l-glutamine, and 2% glucose to obtain 1 × 10^6^ cells/ml. One hundred microliters of this fungal suspension was added to a 96-well plate. Subsequently, 100 μl of MK58911-NH_2_ (15.625 μg/ml [1/2× MIC], 31.25 μg/ml [MIC], and 62.50 μg/ml [2× MIC]), amphotericin B (0.125 μg/ml [MIC]), and fluconazole (1 μg/ml [MIC]), all diluted in RPMI 1640 medium, was added to the plate. The fungal suspension and RPMI 1640 medium were used as the positive and negative controls, respectively. The plate was incubated at 37°C at 150 rpm for 4 h. After that, 10 μl of calcofluor white was added to reach a 50-μg/ml concentration, and the mixture was incubated for 5 min at room temperature. The samples were centrifuged at 3,500 rpm for 5 min (5810R centrifuge; Eppendorf) and washed with PBS. Next, 200 μl of PBS was added to each well, and the images were acquired with an In Cell Analyzer 2000 microscope (GE). The quantification of the average fluorescence intensity per cell to verify the action of the peptide on the chitin content present in the cell wall and the fungal cell count were performed by using In Cell Investigator software. Three independent experiments were performed.

### Cellular and capsule sizes.

The C. neoformans suspension and treatment preparation with the MK58911-NH_2_ peptide were carried out as described above for the cell wall assay. After 4 h, fungal cells were centrifuged and washed with PBS. A sample from each group (control and MK) was suspended in India ink, and images were acquired in a Primovert inverted microscope (Carl Zeiss). Cellular and capsular sizes of parent cells were measured using ImageJ software. Another sample from each group was incubated with 10 μg/ml of capsule-binding mouse monoclonal antibody 18b7 at 37°C at 150 rpm for 1 h. Subsequently, 1 μl of anti-mouse IgG-FITC was added, and the samples were incubated at 37°C at 150 rpm for 30 min. The images were acquired with an In Cell Analyzer 2000 microscope (GE). The quantification of the average fluorescence intensity per cell to verify the action of the peptide on the capsule was performed with In Cell Investigator software. Three independent experiments were performed.

### Zebrafish infected with C. neoformans.

A zebrafish embryo infection model by immersion was used, as previously described for C. albicans, with some modifications (22). The housing and breeding of the fish used in this study received appropriate institutional approvals (permit number 01.0082.2014). Briefly, 15 zebrafish embryos at 24 h postfertilization (hpf) and cultured in embryo medium at 28°C were used. Embryos were coincubated in wells with C. neoformans at 1 × 10^6^ cells/ml by immersion. The plate was incubated at 28°C at 80 rpm for 24 h. Subsequently, the embryos were washed twice with embryo medium and transferred to a 96-well plate (1 embryo/well). Next, 100 μl of MK58911-NH_2_, amphotericin B, and fluconazole, all diluted in embryo medium and at the MIC, was added to the wells. The plate was incubated at 28°C at 80 rpm for 24 h. After that, the embryos were lysed individually in 2% Triton X-100–PBS using a 26-gauge needle. A 1-fold dilution was performed in PBS, and 10 μl was plated onto Sabouraud dextrose agar containing 50 mg/liter chloramphenicol. The plates were incubated at 37°C for 48 to 72 h, and CFU were determined. The groups were tested in triplicate, and two independent experiments were performed. Images of some embryos 4 h after infection with C. neoformans, labeled with calcofluor white, were acquired with an In Cell Analyzer 2000 microscope (GE).

### Antifungal activity.

Anti-Cryptococcus activity was determined by the broth microdilution method according to document M27-A3 from the Clinical and Laboratory Standards Institute (47). Briefly, 100 μl of a fungal suspension at 1 × 10^6^ to 5 × 10^6^ cells/ml in RPMI 1640 medium was added to each well of the 96-well plate. Subsequently, 100 μl of MK58911-NH_2_, MK58911(1–7)-OH, MK58911(8–14)-NH_2_, MK58911(4–11)-OH, and MH58911 (0.48 to 250 μg/ml); amphotericin B (0.016 to 8 μg/ml); and fluconazole (0.063 to 32 μg/ml), all diluted in RPMI 1640 medium, was added to the plate. The fungal suspension and RPMI 1640 medium were used as the positive and negative controls, respectively. The plate was incubated at 37°C at 150 rpm for 48 h. Next, fungal growth was visually observed, and the MIC was determined. Three independent experiments were performed.

### Cytotoxicity.

The impact of peptides on the viability of pulmonary fibroblasts (MRC5) was also evaluated. Cells were cultured in Dulbecco’s high-glucose modified Eagle medium (DMEM; Gibco, Thermo Fisher Scientific) supplemented with 10% fetal bovine serum (FBS) in 25-cm^2^ flasks. For the assay, cells were seeded into 96-well plates at 5 × 10^4^ cells/well. The cells were left to grow at 37°C in a 5% CO_2_ incubator for 24 h until they reached confluence. Medium from the wells was then aspirated, and 100 μl of the MK58911-NH_2_, MK58911(1–7)-OH, MK58911(8–14)-NH_2_, MK58911(4–11)-OH, and MH58911-NH_2_ (0.48 to 250 μg/ml), all diluted in DMEM, was added to the plate. DMEM and dimethyl sulfoxide (DMSO) were used as the positive and negative controls, respectively. The plates were then incubated at 37°C in a 5% CO_2_ incubator for 24 h. Resazurin (0.01%; Sigma-Aldrich) was then used to assess the viability of the cells according to the manufacturer’s instructions. After 4 h of incubation at 37°C in a 5% CO_2_ incubator, the fluorescence intensity was measured using a microplate reader (BioTek) at 570 to 600 nm. The fluorescence values were normalized by the positive control and expressed as a percentage of metabolic activity. Values for the 50% inhibitory concentration (IC_50_) were calculated. Three independent experiments were performed.

### Intramacrophage activity.

The infection assay of Cryptococcus in J774 macrophages was performed according to methods described in a previous study (48), with some modifications. Macrophages (5 × 10^5^ cells/ml) were seeded into a 96-well plate in DMEM supplemented with 10% FBS at 37°C in 5% CO_2_. After 18 to 24 h, they were activated for 1 h with 150 ng/ml phorbol 12-myristate 13-acetate (PMA; Sigma-Aldrich) in serum-free DMEM (SF-DMEM). During that time, 1 × 10^6^ cells/ml of C. neoformans were opsonized with mouse monoclonal antibody 18b7. Next, macrophages were infected with opsonized fungal cells (multiplicity of infection [MOI] of 5:1) for 2 h at 37°C with 5% CO_2_. After that, the macrophages were washed 4 times with PBS. The supernatant was discarded, and 100 μl of the solutions of MK58911-NH_2_, MH58911-NH_2_, amphotericin B, and fluconazole, all diluted in SF-DMEM and at the MIC, was added to the plate. The medium was used as a positive control. The plate was incubated at 37°C in 5% CO_2_ for 24 h. Subsequently, the macrophages were lysed using sterile water for 30 min at 37°C. An aliquot (10 μl) of the 2-fold dilution in PBS was plated onto Sabouraud dextrose agar. The plates were incubated at 37°C for 48 to 72 h, and the CFU were determined. Three independent experiments were performed. The impact of peptides on the viability of macrophages (J774) was also evaluated as described above for MRC5 cells.

### Antibiofilm activity.

The biofilm formation of C. neoformans and the activity of MK58911-NH_2_ and MH58911-NH_2_ were evaluated according to methods described previously (13), with slight modifications. Briefly, C. neoformans was grown in Sabouraud dextrose broth at 30°C at 150 rpm for 24 h. To evaluate the effect of peptides on biofilm formation, a fungal suspension of 1 × 10^7^ cells/ml in minimal medium (20 mg/ml thiamine, 30 mM glucose, 26 mM glycine, 20 mM MgSO_4_·7H_2_O, 58.8 mM KH_2_PO_4_) was prepared, and 100 μl of the suspension was added to individual wells of 96-well polystyrene plates (TPP, Switzerland). Subsequently, 100 μl of MK58911-NH_2_, MH58911-NH_2_ (31.25 to 1,000 μg/ml), amphotericin B (0.25 to 8 μg/ml), or fluconazole (1 to 32 μg/ml), all diluted in minimal medium, was added to the wells. The plate was incubated at 37°C for 48 h. To evaluate the effect of the peptides on the mature biofilm, 100 μl of the fungal suspension was added to individual wells of a 96-well plate, and the plate was incubated at 37°C for 48 h. After that period, the supernatant was removed, and the peptides and drugs were added. The plate was again incubated at 37°C for 48 h. The metabolic activity of the biofilms was quantified by the 2,3-bis(2-methoxy-4-nitro-5-sulfophenyl)-5-[(phenylamino)carbonyl]-2*H*-tetrazolium hydroxide (XTT) reduction assay. For this, a solution with 50 μl of XTT (1 mg/ml in PBS) and 4 μl of menadione (1 mM in ethanol; Sigma-Aldrich, São Paulo, SP, Brazil) was added to each well of the 96-well plate. After 3 h of incubation at 37°C, the fluorescence intensity was measured using a microplate reader (BioTek) at 490 nm. Subsequently, the percentage of metabolic activity of the biofilm treated with peptides/drugs was compared to that of the untreated control. Three independent experiments were performed.

### Statistical analysis.

Graphs and statistical analyses were performed using GraphPad Prism 5.0 (GraphPad Software Inc., La Jolla, CA). The data for the cell wall, fungal cell counts, and CFU (zebrafish and macrophage) were compared using one-way analysis of variance (ANOVA) followed by Dunnett’s correction. The data for cellular and capsule sizes were compared by a *t* test. *P* values of <0.05 were considered significant.
